# Effect of transcutaneous electrical nerve stimulation with 2% lignocaine in dental extraction

**DOI:** 10.6026/973206300210014

**Published:** 2025-01-31

**Authors:** Nilofar Abadul Jabbar, Saravanan Kandasamy, Tharani P., Indra Kumar Periyasamy, Narendar Ramesh, Amudha S.

**Affiliations:** 1 Department of Oral and Maxillofacial Surgery, Vivekanandha Dental College for Women, Tiruchengode, India; 2 Department of Oral and Maxillofacial Surgery, Vinayaka Mission's Sankarachariyar Dental College, Salem, India; 3 Department of Periodontology, Vivekanandha Dental College for Women, Tiruchengode, India

**Keywords:** 2% lignocaine, transcutaneous electrical nerve stimulation, local anesthesia, visual analog scale, prick test, pain

## Abstract

The use of a visual analogue scale to compare and assess the pain, comfort and efficacy of transcutaneous electric nerve stimulation (TENS) with 2% lignocaine during extraction is of interest. Hence, this study was conducted with 20 patients where 10 patients underwent extraction under transcutaneous electrical nerve stimulation and the other 10 patients with 2% lignocaine. Pain and anaesthetic assessment was performed. Transcutaneous electrical nerve stimulation is non-invasive and safe to use, making it far more effective in removing dental injection-related anxiety.

## Background:

The fear and anxiety that patients experience in the dental office, especially with dental injections, which are also referred to as "needle-phobia" or "blenophobia", is the most unpleasant element of being a patient [1]. Inhalation sedation has also been used in place of injectable local anesthetics during dental operations. Nitrous oxide/oxygen is the most commonly used inhalation anesthetic in dentistry. Although nitrous oxide/oxygen has some analgesic properties, its potency (high minimal alveolar concentration) makes it unsuitable for usage in substitution of local anesthetics since it may not always have the desired effects on all individuals. The equipment and logistics of safe delivery, such as operatory space, equipment charges, supply costs and patient costs, are further disadvantages of nitrous oxide. Thus, the goal was to provide a secure and efficient substitute for inhalation sedation and injectable local anesthetics that could be utilized in dental offices [2]. Electronic dental anesthesia (EDA) or transcutaneous electrical nerve stimulation has been utilized to help manage pain in adults and children in recent years. Transcutaneous electrical nerve stimulation is a technology based on the well-established pain control hypothesis put forth by Malzack and Wall in 1965 that offers a promising low current dental anesthetic delivery approach. It is widely utilized to relieve pain from a variety of illnesses, including endometriosis, arthritis, sports injuries, multiple sclerosis, fibromyalgia, severe diabetic neuropathy and spinal cord injury, in many medical and paramedical professions [3]. There is still a lack of research on the use of transcutaneous electrical nerve stimulation as an anesthetic device, even though Shane and Kessler initially reported its usage in dentistry in 1967. Patients can accept it broadly and it's safe and non-invasive. Transcutaneous electrical nerve stimulation has been shown to be an effective way to manage discomfort during some dental operations and to offer a considerable improvement over other common local anesthetic therapies [4]. According to Pashley *et al.* pain during needle injection is caused by administering the anesthetic fluid too quickly or forcefully. Furthermore, the flow rate and pressure rate cannot be properly controlled with manual conventional syringes, leading to uneven and uncomfortable injections. Reducing the pain and suffering related to the administration of local anesthesia might improve the patient's comfort and happiness. In actuality, the primary objective of any physician is to provide local anesthetic without causing pain [5]. Therefore, it is of interest to clinically evaluate the efficiency of transcutaneous electrical nerve stimulation in extraction technique as a prospective substitute for injectable local anesthetics in geriatric patients, in order to provide a realistic alternative in the dentist's pain control arsenal.

## Materials & Methods:

This comparative prospective study was carried out in the department of oral and maxillofacial surgery at Vivekananda Dental College for Women in the year 2022. The study was approved by the institutional ethical committee prior to the study (No: VDCW/IEC/308/2022). Every single patient was informed about the surgical technique and gave their informed consent. Study comprised of 20 patients in total, where it was divided into two groups with 10 patients in each group. The patients' lots, which served as a straightforward random sampling technique, divided the two extraction groups. Ten patients from Group A and ten patients from Group B, respectively, received extractions using 2% lignocaine and transcutaneous electrical nerve stimulation, respectively. It took us two months to complete our study session. For every patient, a proforma comprising name, age, sex, address, chief complaint, medical history, prior dental history, intraoral examinations and past medical history was employed. Adult patients aged 45-50 year old who are healthy and cooperative with grade III mobility on clinical examination were included in our study. We excluded the patients who are under corticosteroids, having systemic illness like heart disease, cardiac pacemakers, seizure disorders and neurologic disorders. The flowchart of the study is explained in (Figure 1 - see PDF).

The materials used here were two channel Transcutaneous Electrical Nerve Stimulator T.E.N.S. marketed by Sheetla-Tec Industries (An ISO 9001: 2015 Certified Company) (Figure 2 - see PDF), Haryana, Electrode gel (Figure 3 - see PDF), 25 Gauge needle and Lignocaine 2%. Figure 4 (see PDF) shows the electrode pads and control unit that come with the T.E.N.S. Two separate galvanic channels make up its composition. A fixed pulse rate and amplitude control of up to 220 mA can be achieved by each channel, which is powered by a 9 V battery with frequencies ranging from 2 Hz to 150 Hz. Each channel also has a frequency control knob.

## Outcome measures:

The primary outcome of our research was to assess and contrast the pain following extraction using transcutaneous electrical nerve stimulation and 2% lignocaine. A visual analog scale was used to measure the degree of pain. Patients were asked to rate their level of pain on a scale of 0 to 10. Here, representation of a cheerful patient at one end and a sobbing patient at the other represent 0 and 10, respectively, representing "absolutely no pain" and "the worst pain imaginable", Using a prick test, the secondary outcome evaluates the anesthetic impact of transcutaneous electrical nerve stimulation and 2% lignocaine. Pricking the patient's marginal gingiva, connected gingiva and the area around the tooth allowed researchers to determine the anesthetic impact. Patients were then asked to raise their hands if they felt any discomfort.

## Intervention:

Before starting the actual treatment, the patient was given a brief explanation of the method and the electrode pad placement was determined. The electrode pad implantation location was gently cleansed with surgical spirit to remove any oils or anything that would impede the flow of current. Electrode gel was added to the electrode pads prior to placement. Once the electrode pads were in position, the patient was told to maintain their lips open during the procedure. Surgical tape was used to hold the electrode pads firmly in place and prevent them from moving. After turning on the device, the researcher increased the amplitude knob to progressively increase the patient's level of electronic anesthesia until a discernible sensation began to occur. In order to give the patient time to get used to the new sensation of electronic anesthesia, this amplitude level was kept at this level for 20 seconds. After then, the cycle was repeated with increasing amplitude until quivering or fasciculation was noticed in close proximity to the pads. The lower eyelid and upper lip twitched in cases involving the maxillary arch, whereas the lower lip twitched in cases involving the mandibular arch. This was the lowest feasible level, called the "therapeutic level of stimulation" and the treatment could continue at it. If there was pain or discomfort felt by the patient during the process, the amplitude was gradually increased to "dail-out discomfort" within an acceptable range. When experiencing any discomfort, the patient was instructed to raise their hand and the transcutaneous electrical nerve stimulation amplitude was decreased while the researcher adjusted the stimulation intensity. Following the procedure, all controls were returned to their initial state of zero. After turning off the equipment, the connections were eventually cut.

## Results:

The statistical package for social science (SPSS), version 25 was used for all of the statistical analysis. The samples were examined to see if they were regularly distributed using the Kolmogorov-Smirnov test. Furthermore, the outcome of the histogram was uneven around the mean of the distribution, leading us to conclude that the data were not normally distributed. The data were shifted to the left, as indicated by their negative kurtosis. Thus, the following resulted from doing the Mann-Whitney U test (Table 1). 10 patients had undergone extraction under transcutaneous electrical nerve stimulation, out of which 4 were male patients and 6 were female patients and 10 patients had undergone extraction under 2% Lignocaine out of which 4 were male patients and 6 were female patients (Table 2 & Table 3). The amount of pain experienced throughout the surgery was measured using the visual analog scale. Patients' proposed Visual Analog Scale scores fell into four categories: 0 represented no pain; 1-3 represented mild pain; 4-6 represented moderate to severe pain; 7-9 represented very severe pain and 10 represented the worst possible pain.

## Discussion:

Pain is the most unpleasant and uncomfortable part of dentistry and it can lead to a patient acting significantly less cooperatively in the dental office. It is distressing that the devices meant to ease patients' pain also exacerbate their discomfort and worry. While transcutaneous electrical nerve stimulation offers the advantages of being non-invasive and safe to use, it is still far more effective than local anesthesia for minor dental procedures in relieving pain associated with injections. The purpose of this study was to better understand the anesthetic effect of transcutaneous electrical nerve stimulation during extractions and to determine whether this technique may replace local anesthesia in permanent teeth extractions of grade III mobility. For this reason, Grade III extractions performed in the Oral and Maxillofacial Surgery department were chosen for this investigation. "Transcutaneous Electrical Nerve stimulation is the direct stimulation of the nerves by short-duration, small amplitude electric pulses," according to All good [6]. Three types of transcutaneous electrical nerve stimulation units are identified. For both acute postoperative pain and chronic temporomandibular joints pain, the most common mode utilized is high-frequency (25-150 Hz) electronic dental anesthesia. When high-frequency transcutaneous electrical nerve stimulation becomes unusable due to accommodation when treating chronic pain, low frequency (2-10 Hz) is employed. The measurement of precise vertical dimension of rest and the treatment of persistent temporomandibular joints pain are two further applications for ultralow-frequency (0.5-2 Hz). In order to prevent adverse skin reactions, dental procedures should only employ a balanced, biphasic wave shape with a zero net DC component [7]. The application of transcutaneous electrical nerve stimulation was founded on a number of interconnected theories about the transmission of pain and how to block these pathways. The first of these theories is the gate control theory proposed by Melzack and Wall [3]. Another explanation for the workings of transcutaneous electrical nerve stimulation is that endorphins are released as a result of electric stimulation. These endorphins attach to opiate receptors and prevent the transmission of painful impulses. According to a different idea, stimulation-induced analgesia involves the creation of dopamine, norepinephrine and serotonin and the analgesic impact of transcutaneous electrical nerve stimulation is directly correlated with a rise in serotonin. It is currently unclear how exactly electronic anesthesia reduces pain, though it may work by combining one or more theories. In the first group, transcutaneous electrical nerve stimulation was used to extract teeth from ten patients. These were mobile teeth of grade III, their roots either intact or resorbed. The mean Visual Analog Scale score for this group was 0.40, meaning that some patients had moderate pain or discomfort during extraction and that the clinician thought the anesthetic was effective. The scale showed p<0.143 to be extremely significant. In contrast, 10 patients in the second group underwent extractions with 2% lignocaine. They were also grade III mobile teeth, their roots either resorbed or undamaged. The Visual Analog Scale data for this group revealed a mean score of 0.00, meaning that not a single patient reported feeling pain or discomfort during the extraction process. Table 4 displays a p-value of 0.000 as indicated by the scale. The conventional Prick test was used to validate both groups. Nonetheless, 75% of the patients showed a positive response to transcutaneous electrical nerve stimulation in comparison with lignocaine. In terms of hemostasis and wound healing, transcutaneous electrical nerve stimulation proved to be more effective for 100% of the patients. According to some hypotheses, transcutaneous electrical nerve stimulation improves patients' comfort after surgery. It looks that there are two ways that this is achieved. Initially, teeth do not have to heal from a blood flow loss caused by injection-based local anesthetic since the blood flow to the treated area is boosted. Second, the enhanced feeling of wellbeing may persist for several hours following the removal of the electrodes due to the production of endogenous opioids, such as endorphins and enkephalins. None of our patients experienced skin redness or responses to the electrodes, despite some studies showing transient skin redness over the electrode implantation site as a result of increased blood circulation to that area [8, 9, 10-11].

## Conclusion:

Transcutaneous electrical nerve stimulation is far more effective than local anesthesia for relieving dental injection-related pain as it is safe and non-invasive. Therefore, electronic dental anesthesia transcutaneous electrical nerve stimulation is a useful addition to a dentist's toolkit. The therapeutic effectiveness of these anesthetic modalities will be maximized by accurately utilizing modern technology such as artificial intelligence in electronic dental anesthesia for pain threshold assessment which will soon surpass the need of needles. It should be noted that validation using a large sample size is highly relevant.

## Figures and Tables

**Table 1:** Statistical test

**Ranks**			
**Groups**	**N**	**Mean rank**	**Sum of ranks**
Tens	10	12.5	125
La	10	8.5	85
Total	20

**Table 2:** Total number of patients

**Groups**	**Number of patients**
Group a (tens)	10
Group b (2% lignocaine)	10

**Table 3:** Gender distribution

**Groups**	**Male**	**Female**
Group a (tens)	4	6
Group b (2%)	4	6

**Table 4:** Mean visual analog scale score

	**Number of cases**	**Mean**	**Standard deviation**	**P- value**
Tens	10	0.4	0.516	0.143
2%lignocaine	10	0	0	0
